# Elevated serum IL-21 levels are associated with stable immune status in kidney transplant recipients and a mouse model of kidney transplantation

**DOI:** 10.18632/aging.103713

**Published:** 2020-09-29

**Authors:** Luying Guo, Junhao Lv, Jian Zhang, Hao Deng, Shi Feng, Shuaihui Liu, Pengpeng Yan, Jingyi Zhou, Hui Chen, Meifang Wang, Qin Zhou, Huiping Wang, Jianghua Chen, Yu Kuang, Jia Shen, Rending Wang

**Affiliations:** 1 Kidney Disease Center, The First Affiliated Hospital, College of Medicine, Zhejiang University, Hangzhou 310003, Zhejiang, China; 2 National Key Clinical Department of Kidney Diseases, College of Medicine, Zhejiang University, Hangzhou 310003, Zhejiang, China; 3 Zhejiang Provincial Key Laboratory of Kidney Disease Prevention and Control Technology, College of Medicine, Zhejiang University, Hangzhou 310003, Zhejiang, China; 4 Zhejiang University Institute of Nephrology, Hangzhou 310003, Zhejiang, China; 5 The First Affiliated Hospital, College of Medicine, Zhejiang University, Hangzhou 310003, Zhejiang, China; 6 Medical Physics Program, University of Nevada, Las Vegas, NV 89154, USA; 7 Organ Donation and Coordination Office, The First Affiliated Hospital, College of Medicine, Zhejiang University, Hangzhou 310003, Zhejiang, China

**Keywords:** kidney transplantation, IL-21, acute rejection, TCMR, ABMR

## Abstract

Allograft rejection after renal transplantation remains a challenge to overcome. Interleukin (IL)-21, a cytokine with pleiotropic effects, maintains immune homeostasis post-transplantation. Here, we report higher levels of IL-21 in kidney transplant recipients with non-rejection (NR) than in recipients with T cell-mediated rejection (TCMR, *P* < 0.001) and antibody-mediated rejection (ABMR, *P* = 0.005). We observed a negative correlation between IL-21 and creatinine (Cr) levels (*P* = 0.016). The receiving operating characteristic (ROC) curve showed a promising diagnostic value of IL-21 to identify acute rejection with an area under the curve (AUC) of 0.822 (*P* < 0.001). In contrast, exogenous administration of IL-21 accelerated acute rejection in a comparative translational kidney transplant (KT) mouse model. Reduced IL-21 levels in the peripheral blood were observed in KT mice after IL-21 injection. Further analysis revealed that increased IL-21 levels in the spleen induced proliferation of CD4+ T cells and CD19+ B cells after IL-21 treatment. Our findings suggest a critical function of IL-21 in kidney transplantation and the potential involvement of the IL-21/IL-21R pathway in acute rejection management.

## INTRODUCTION

Allograft rejection remains one of the major barriers to the long-term survival of the graft. More potent immunosuppressive drugs and better histocompatibility between recipients and donors have altered the characteristics of allograft rejection. The risk of rejection within 1 year after kidney transplantation has declined to less than 15% [1]. However, the overall graft survival rates have still not improved due to the intractability of rejection [2]. Based on different clinicopathologies, rejection could be divided into T cell-mediated rejection (TCMR) and antibody-mediated rejection (ABMR) [1]. Patients with emerging rejection face an increased risk of graft failure, especially ABMR [3, 4].

Failure of immunosuppression and activation of the immune system against the allograft in recipients are majorly responsible for its rejection [1, 5]. The involvement of innate and adaptive immunity in TCMR and ABMR has been demonstrated by numerous clinical and animal studies [6–8]. Apart from the signals transduced by exogenous antigens and costimulatory molecules, cytokines secreted by immune cells, identified as “signal 3”, can activate the immune system, particularly T and B cells [9, 10]. Interleukin (IL)-21 is an important “signal 3” molecule that exerts its effects on T and B cells via autocrine and paracrine signaling.

IL-21 is a proinflammatory cytokine involved in several immune dysfunction diseases. It is one of the main cytokines secreted by T follicular helper (Tfh) cells [11, 12], which stimulate the differentiation of B cells into antibody-secreting plasma cells and have been reported to be involved in autoimmune disorders [13]. Weinstein et al. revealed the successive secretion pattern of IL-21 and IL-4 by Tfh cells in germinal centers (GCs) using a double reporter mouse model [14] and highlighted the involvement of IL-21 in T and B cell crosstalk. Moreover, genome-wide association studies and animal studies have demonstrated a high expression of IL-21 in patients with autoimmune diseases, such as systemic lupus erythematosus [15, 16], rheumatoid arthritis [17], type 1 diabetes [18], and celiac disease [19]. The study of early onset of inflammatory bowel disease in patients deficient for IL-21 [20] has advanced the understanding of IL-21 as a proinflammatory cytokine.

Increased levels of IL-21 in hyperplastic follicles were observed in a rhesus macaque kidney transplant model with ABMR [9]. The antagonist of IL-21 receptor (IL-21R) was reported to block B cell differentiation [21]. Another study reported that the blocking of IL-21R ameliorated allograft rejection in a mouse skin transplant model [22]. Further, the role of IL-21 in immune tolerance in kidney transplant recipients is dependent on granzyme B (GZMB)^+^ B cells [23].

Previous studies have primarily focused on the effect of IL-21R blocking on transplantation or its *in vitro* effects. In the present study, we demonstrated the involvement of IL-21 in acute rejection after kidney transplantation. A unique cytokine profile was identified in patients with or without acute rejection after kidney transplantation. Surprisingly, higher levels of IL-21, accompanied by a comparable class switching rate of antibodies, were closely related with a stable immune status in patients after transplantation. To further directly investigate the function of IL-21 in maintaining a stable immune status, we generated a mouse model of kidney transplant (KT) with tail vein injection of exogenous IL-21. Intriguingly, histopathological analysis revealed emerging acute rejection with an increased number of B cells after injection of exogenous IL-21. We believe that IL-21 can serve as a potential therapeutic target for acute rejection.

## RESULTS

### Patient characteristics

The demographic characteristics of different subgroups are summarized in Table 1. There was no difference among non-rejection (NR), TCMR, and ABMR cohorts regarding the donor-associated parameters, recipient’s age, primary kidney disease, dialysis, delayed graft function (DGF) rate, or HLA-mismatch (HLA-MM). Considerable differences in recipient’s gender (*P* = 0.025) and induction (*P* = 0.001) were noted among the three cohorts.

**Table 1:** Demographics of patients.

	**NR (N=30)**	**AR (N=36)**	**TCMR (N=28)**	**ABMR (N=8)**	***P^a^***	***P^b^***
**Donor associated parameters**						
Age (years)	43.47±2.66	45.33±1.90	45.39±2.18	45.13±4.12	0.844	0.162
Serum creatinine (μmol/L)	92.36±11.48	99.19±12.98	101.03±14.62	92.75±29.97	0.892	0.286
Donor type (DCD/LD)	19/11	22/14	19/9	3/5	0.291	0.853
Donor sex(M/F)	22/8	24/12	19/9	5/3	0.807	0.557
**Recipients associated parameters**						
Age (year)	41.27±1.88	36.94±1.79	38.00±2.08	33.25±3.38	0.511	0.791
Sex (M/F)	10/20	22/14	16/12	6/2	0.054	0.025
Kidney primary disease					0.697	0.466
Chronic kidney disease	25	25	20	5		
IgA nephropathy	1	1	1	0		
Lupus nephritis	0	1	1	0		
Polycystic kidney disease	0	3	2	1		
Diabetic nephropathy	0	1	1	0		
Others	4	5	3	2		
Dialysis	0.495	0.480
Hemodialysis	20	20	14	6		
Peritoneal dialysis	10	15	13	2		
None	0	1	1	0		
DGF	1/29	31/5	5/23	0/8	0.100	0.137
Induction	0.004	0.001
Simulect	9	19	13	6		
ATG	5	13	11	0		
Rituximab	0	1	1	2		
None	16	3	3	0		
HLA-MM	2.63±0.23	2.61±0.22	2.68±0.27	2.38±0.26	0.837	0.924
Stable creatinine	92.36±11.48	149.03±16.04	138.75±10.48	185.00±34.18	0.011	0.324
Highest creatinine	92.36±11.48	248.25±24.37	235.75±25.80	292.00±63.42	<0.001	0.004
DSA	0	8	0	8	0.006	<0.001
Time of rejection	NA	575.14±114.03	440.57±103.28	1046.13±336.15	NA	NA

NR, no rejection; AR, acute rejection; TCMR, T cell mediated rejection; ABMR, antibody mediated rejection; DGF, delayed graft function; ATG, anti-thymocyte globulin; HLA-MM, HLA-mismatch; DSA, donor specific antibody; *P^a^*, *P* value represents the difference between NR and AR; *P^b^*, *P* value represents the difference between NR, TCMR, and ABMR, using one-way ANOVA.

### Higher peripheral blood IL-21 levels in stable transplant recipients

**Figure 1:**
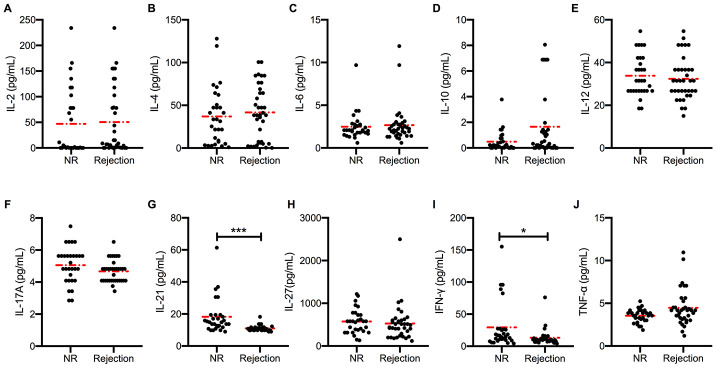
**Higher peripheral blood IL-21 levels in stable transplant recipients.** Absolute concentration (pg/mL) of serum IL-2, IL-4, IL-6, IL-10, IL-12, IL-17, IL-21, IL-27, IFN-γ, and TNF-α (**A**–**J**) in recipients with (*n* = 36) and without (*n* = 30) active rejection after kidney transplantation. Red dash dot line represents the mean value. *P < 0.05 and **P < 0.01 compared with NR cohorts. IL, interleukin; IFN, interferon; NR, non-rejection; TNF, tumor necrosis factor.

The serum cytokine levels of recipients with or without acute rejection after kidney transplantation were measured using the Luminex assay. Levels of major proinflammatory cytokines, such as IL-2, IL-4, and IL-6 (Figure 1A–1C), were slightly higher in acute rejection patients than in immune stable patients after kidney transplantation. Unexpectedly, the levels of IL-21, which is widely considered as a driving factor of GC B cell differentiation, were lower in acute rejection patients (11.02±0.29 pg/mL) than in immune stable patients (18.21±2.00 pg/mL, *P* < 0.001) (Figure 1G). Similarly, the circulating levels of interferon (IFN)-γ in acute rejection patients (13.18±2.02 pg/mL) were lower than in immune stable patients (29.41±6.59 pg/mL, *P* = 0.012) (Figure 1I). Comparable levels of IL-10, IL-12, IL-17, IL-27, and tumor necrosis factor (TNF)-α (Figure 1D–1F, 1H, and 1J) were observed between recipients with and without acute rejection.

### Distinct circulating cytokine levels in different types of rejection

**Figure 2:**
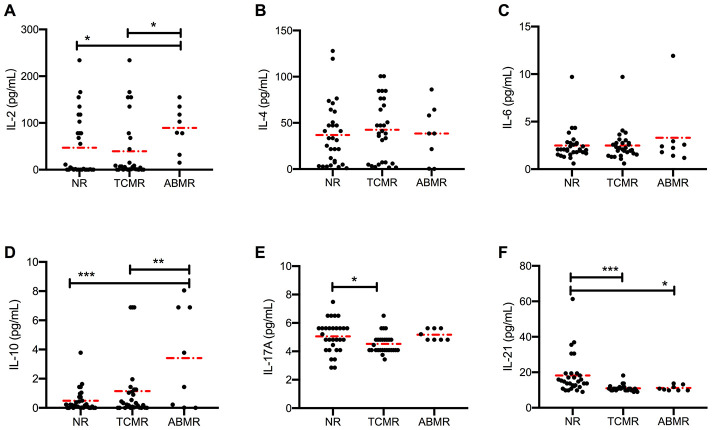
**Distinct circulating cytokine levels in different types of rejection.** Serum levels of IL-2, IL-4, IL-6, IL-10, IL-17, and IL-21 (**A**–**F**) in patients in NR (*n* = 30), TCMR (*n* = 28) and ABMR (*n* = 8) cohorts were measured before anti-rejection therapy. Dash dot line represents the mean value. *P < 0.05, **P < 0.01, and ***P < 0.001. IL, interleukin; ABMR, antibody-mediated rejection; NR, non-rejection; TCMR, T cell-mediated rejection.

To further characterize and compare acute rejection subgroups, we divided the acute rejection cohort into TCMR and ABMR cohorts based on the histological evidence provided by Banff in 2017 [24]. We found that serum IL-2 levels were higher in the ABMR cohort than in the NR (*P* = 0.014) or TCMR (*P* = 0.016) cohort (Figure 2A). By contrast, serum levels of IL-4, IL-6 (Figure 2B and 2C), IL-12, IL-27, and TNF-α (Supplementary Figure 1A–1C) showed no detectable difference among the three cohorts. The IL-21 levels in the NR cohort were higher than in both TCMR (10.98±0.35 pg/mL, *P* < 0.001) and ABMR (11.15±0.56 pg/mL, *P* = 0.008) cohorts (Figure 2F). However, no difference was found between the two rejection subgroups. Elevated levels of IL-10 were present in ABMR cohort, and slightly reduced levels of IL-17 and IFN-γ were present in the TCMR cohort (Figure 2D, 2E and Supplementary Figure 1D).

**Figure 3:**
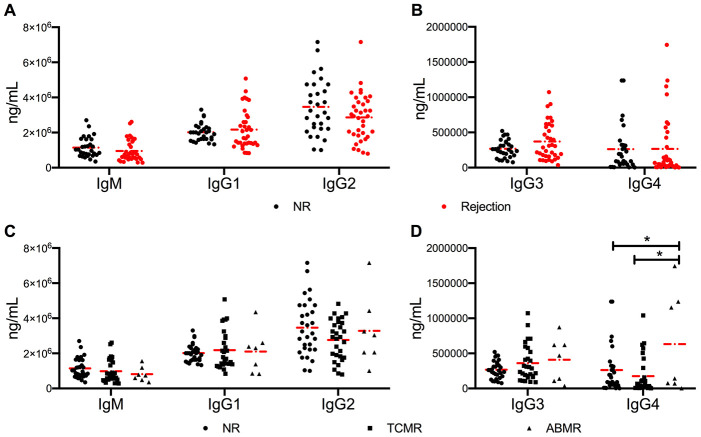
**No significant difference in antibody class switching between NR and active rejection cohorts was observed.** (**A**, **B**) Levels of IgM and IgG1-4 in NR (n =30) and rejection (n = 35) groups. (**C**, **D**) Levels of IgM and IgG1-4 in NR (n =30), TCMR (n = 28), and ABMR (n = 7) cohorts. The dash dot line represents the mean value. The difference among the three subgroups was analyzed using one-way ANOVA. *P < 0.05. ABMR, antibody-mediated rejection; ANOVA, analysis of variance; NR, non-rejection; TCMR, T cell-mediated rejection.

Cytokines are characterized as “signal 3” in T and B cell crosstalk, which promotes B cell differentiation, maturation, and class switching. The class switching rate of B cells was measured by detecting the levels of serum immunoglobulins. No difference was found in the levels of IgM and IgG1-3 in the three cohorts (Figure 3A–3C). The circulating levels of IgG4 were high in the ABMR cohort (*P* = 0.044 vs. NR cohort and *P* = 0.012 vs. TCMR cohort) (Figure 3D).

### Serum IL-21 levels negatively correlate with allograft function

**Figure 4:**
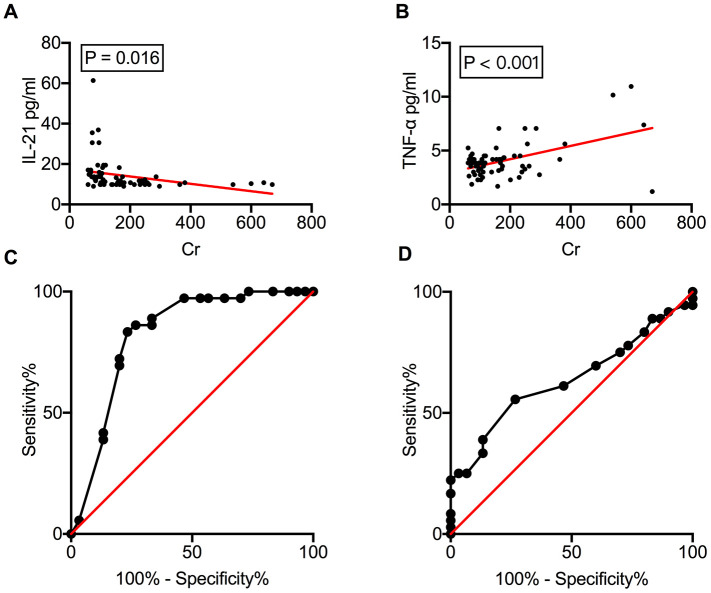
**Serum IL-21 levels correlate with allograft function.** Correlation of serum IL-21 and TNF-α levels with serum Cr levels (**A**, **B**). The ROC curves of IL-21 and TNF-α to investigate their ability to distinguish between rejection and NR recipients (**C**, **D**). Cr, creatinine; IL, interleukin; NR, non-rejection; ROC, receiver operating characteristic; TNF, tumor necrosis factor.

The correlation between circulating cytokine levels and allograft function was analyzed (Supplementary Table 1). Linear regression analysis revealed a good correlation of serum IL-21 (*ρ* = −0.018, *P* = 0.016) and TNF-α (*ρ* = 0.006, *P* < 0.001) (Figure 4A and 4B) levels with serum creatinine (Cr), indicating that circulating cytokines could be a potential biomarker for monitoring renal dysfunction. The diagnostic capacity of IL-21 and TNF-α in distinguishing allograft rejection was measured using the receiver operating characteristic (ROC) curve. The area under the curve (AUC) for serum IL-21 levels was 0.822 (*P* < 0.001), with sensitivity and specificity of 72.22% and 80%, respectively, and a cut-off value of 11.47 pg/mL (Figure 4C). TNF-α exhibited less diagnostic capacity in rejection diagnosis with an AUC of 0.636 (*P* = 0.059) (Figure 4D).

### Exogenous IL-21 promotes allograft rejection in mice

**Figure 5:**
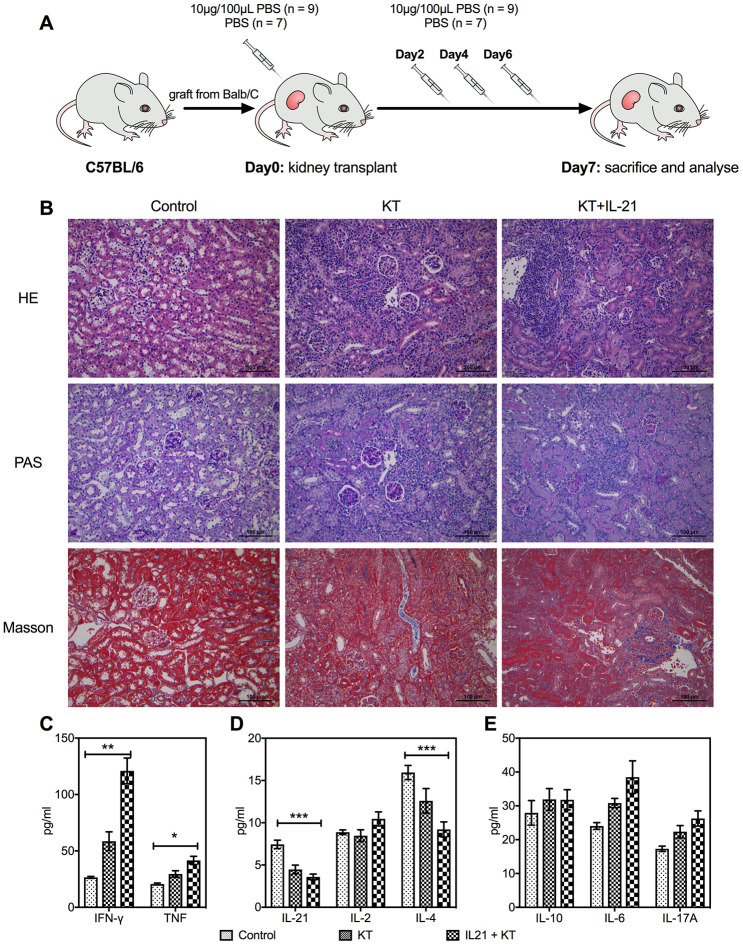
**Exogenous IL-21 promotes inflammatory infiltration in the allograft.** C57BL/6 mice received renal allografts from Balb/c mice. Kidney recipients were injected recombinant murine IL-21 (10 μg in 100 μL PBS; *n* = 9) or 100 μL PBS (*n* = 7) in the tail vein on days 0, 2, 4, and 6 after transplantation (**A**). Blank control (*n* = 3) of mice without transplantation received tail vein injection of PBS simultaneously. All animals were sacrificed 7 days post-transplantation. Histologic evaluation of kidney allografts from Control, KT, and IL-21+KT recipients on day 7 post-transplantation stained with (**B**) H&E, PAS, and Masson’s stains (scale bar: 100μm). Levels of (**C**) IFN-γ and TNF, (**D**) IL-21, IL-2, and IL-4, (**E**) IL-10, IL-6, and IL-17 in the transplanted kidney of KT subgroups and primary kidney of Control are shown. Values represent mean (±SEM). *P < 0.05, **P < 0.01, and ***P < 0.001. H&E, hematoxylin and eosin; IL, interleukin; PAS, periodic acid–Schiff; PBS, phosphate-buffered saline; KT, kidney transplant; SEM, standard error of the mean.

To explore the function of IL-21 in transplant immunobiology, we generated KT mice with tail vein injection of exogenous IL-21 on days 0, 2, 4, and 6 post-transplantation (Figure 5A). Hematoxylin and eosin (HE), periodic acid-Schiff (PAS), and Masson’s staining revealed enhanced infiltration of inflammatory cytokines and fibrosis in the allograft that worsened with IL-21 administration (Figure 5B). Next, the cytokine levels in the transplanted kidney were estimated; similar to the inflammatory infiltration, the expression of proinflammatory cytokines such as IFN-γ (*P* < 0.001 vs. control group and *P* = 0.008 vs. KT group) and TNF-α (*P* = 0.011 vs. control group) was upregulated (Figure 5C). The IL-21 (*P* < 0.001 vs. control group) and IL-4 (*P* = 0.011 vs. control group) levels in the transplanted kidney reduced (Figure 5D). No difference was found in the levels of IL-10, IL-6, and IL-17 in the transplanted kidney among the three groups (Figure 5E).

**Figure 6:**
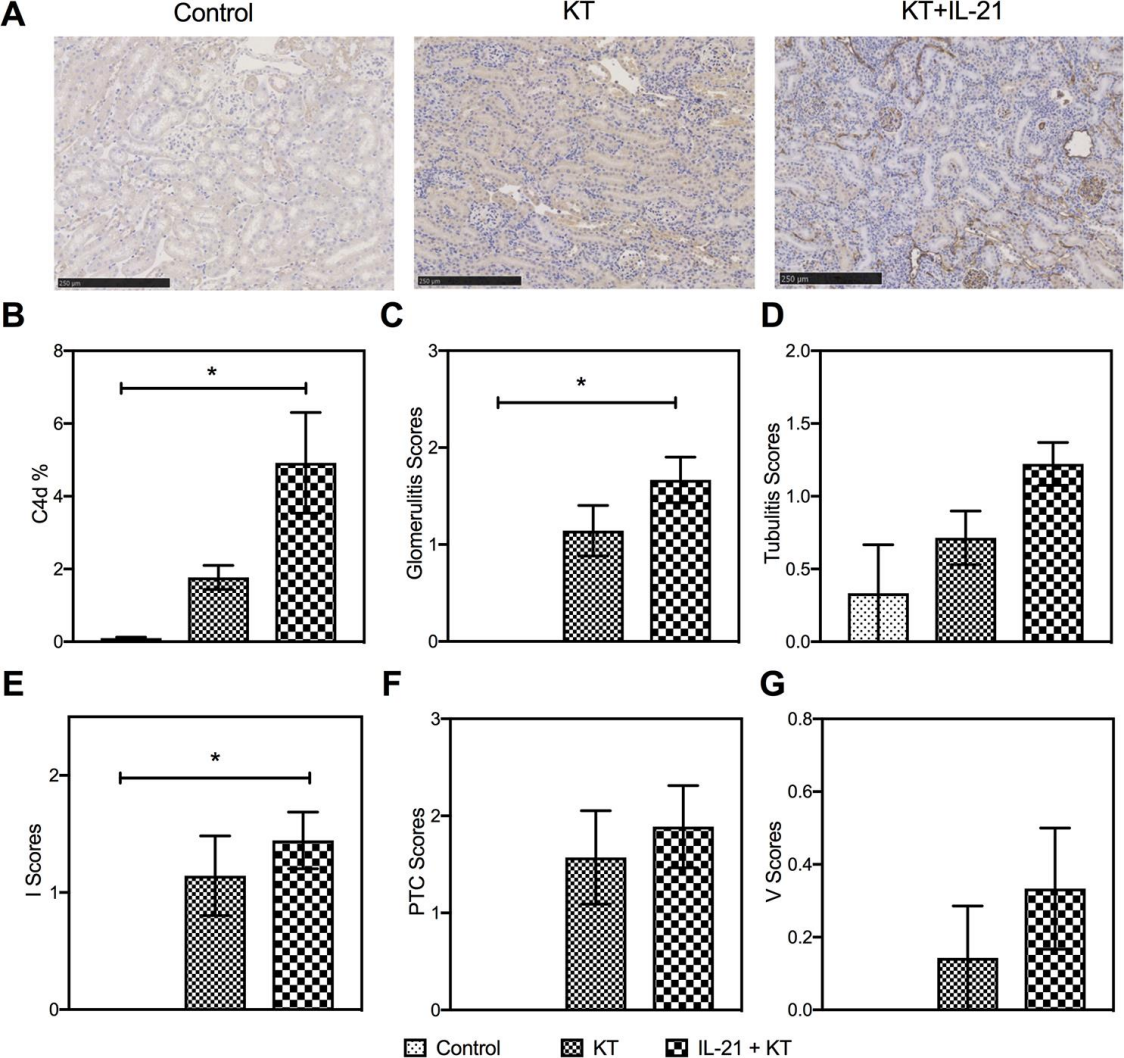
**Exogenous IL-21 promotes active kidney rejection in mice.** (**A** and **B**) Expression of C4d in the allograft (scale bar: 250μm). Histopathology scores of glomerulities, tubulitis, I, PTC, and V (**C**–**G**) of the kidney allografts from Control, KT, and IL-21+KT recipients. Values represent mean (±SEM). *P < 0.05, **P < 0.01, and ***P < 0.001. PTC, peritubular capillaritis; I, interstitial inflammation; V, intimal arteritis; IL, interleukin; KT, kidney transplant; SEM, standard error of the mean.

Next, we evaluated the histopathology of the allograft. A heavy deposit of linear C4d in peritubular capillaries (*P* = 0.016 vs. control group) after IL-21 injection (Figure 6A and 6B) was observed. More severe allograft injury, including glomerulitis, tubulitis, and interstitial inflammation, was observed in KT mice with IL-21 treatment (Figure 6C, 6D, and 6E). Peritubular capillary and intimal arteritis showed no difference (Figure 6F and 6G). The donor-specific antibody (DSA) titers in the kidney recipients were measured by flow cytometry. Higher DSA titers were observed after IL-21 administration (*P* = 0.008 vs. control group) (Supplementary Figure 2).

### Exogenous IL-21 alters cytokine and IL-21R expression in mice after kidney transplantation

**Figure 7:**
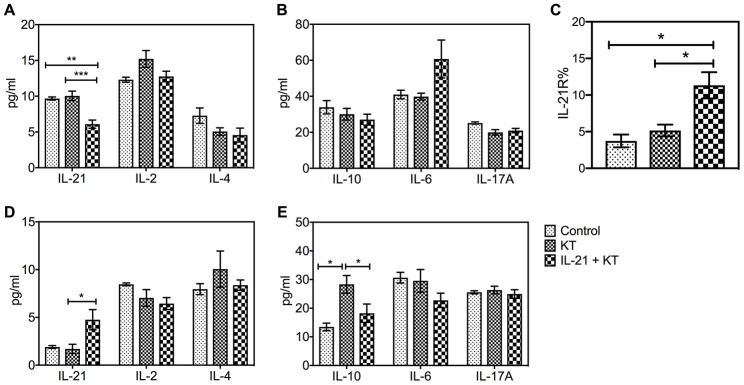
**Exogenous IL-21 alters cytokine and IL-21R expression in mice after kidney transplantation.** The level of IL-21, IL-2, and IL-4, and IL-10, IL-6, and IL-17A in the peripheral blood (**A** and **B**) and spleen (**D** and **E**) on day 7 post-transplantation was measured by CBA. (**C**) Histologic evaluation of IL-21R expression in the spleen of subgroups. Values represent mean (±SEM). *P < 0.05, **P < 0.01, and ***P < 0.001. CBA, cytometric bead array; IL, interleukin; SEM, standard error of the mean.

The cytokine levels in the peripheral blood and spleen of KT mice were measured on the day mice were sacrificed. Interestingly, the peripheral IL-21 levels in mice after administration of exogenous IL-21 were lower than in mice injected with phosphate-buffered saline (PBS) (*P* = 0.004) and control groups (*P* < 0.001) (Figure 7A). Moreover, the IL-6 levels increased after IL-21 treatment, but no major difference was found (Figure 7B). No difference was found among the levels of IL-2, IL-4, IL-10, and IL-17 (Figure 7A and 7B), whereas an increased level of inflammatory cytokine TNF-α was observed (*P* = 0.011 vs. control group) (Supplementary Figure 3A). A substantial increase in IL-21 levels in the spleen was observed after administration of exogenous IL-21 (*P* = 0.040 vs. PBS injection) (Figure 7D), with a higher expression of IL-21R (*P* = 0.021 vs. PBS injection and *P* = 0.011 vs. control group) (Figure 7C and Supplementary Figure 3C–3E). Furthermore, compared with the other two groups, IL-10 levels were higher in the KT group (*P* = 0.018 vs. control group, *P* = 0.014 vs. IL-21+KT group) (Figure 7E).

### Exogenous IL-21 induces B and T cell proliferation

**Figure 8:**
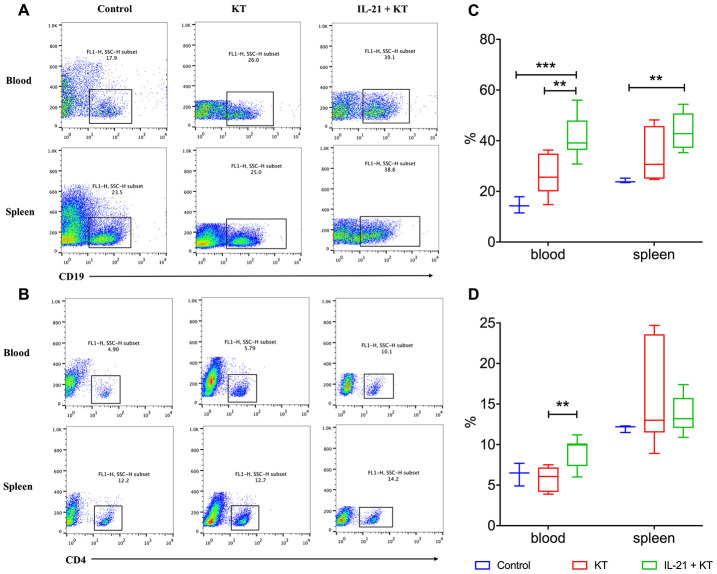
**Exogenous IL-21 induces B cell and T cell proliferation.** Flow cytometry analysis to measure CD19+ B (**A**) and CD4+ T (**B**) lymphocytes in the peripheral blood and spleen of recipients and controls. Distribution of proportions of CD4^+^ T cells (**C**) and CD19^+^ B cells (**D**) of total lymphocytes on day 7 post-transplantation. Boxes and whiskers represent minimum to maximum of all the data. **P < 0.01 and ***P < 0.001 one-way ANOVA. ANOVA, analysis of variance; IL, interleukin.

Flow cytometry was performed to analyze B and T cells in the peripheral blood and spleen. After kidney transplantation, the number of B cells slightly increased, and the number was higher after IL-21 stimulation both in the peripheral blood (*P* < 0.001 vs. control group, *P* = 0.008 vs. KT group) and the spleen (*P* = 0.004 vs. control group) (Figure 8A, 8C). Furthermore, the number of CD4^+^ T cells in the peripheral blood steadily increased in kidney transplanted mice administered IL-21 than in those administered PBS (*P* = 0.003) (Figure 8B, 8D). The number of CD4^+^ T cells was more variable in the spleen, with no major difference (Figure 8B, 8D).

## DISCUSSION

In the present study, we measured the serum levels of IL-21 in acute rejection patients post kidney transplantation and assessed its function in a mouse KT model. Reduced serum levels of IL-21 were detected in both TCMR and ABMR cohorts as compared to immune stable recipients. Serum IL-21 levels negatively correlated with allograft function, showing a promising diagnostic value with an AUC of 0.822 (*P* < 0.001). Interestingly, no difference in class switching was found. Administration of exogenous IL-21 accelerated allograft rejection in KT mice, accompanied by reduced levels of IL-21 in the peripheral blood and enrichment of IL-21 in the spleen. Further analysis revealed IL-21-induced enhanced proliferation of T and B cells in the spleen resulting in acute humoral rejection.

IL-21 is a cytokine with pleiotropic effects; it maintains the immune homeostasis including in patients after solid organ transplantation [25]. It is identified as the most potent plasma cell inducer *in vitro* [26]. IL-21 activates both Tfh cells [15, 27] and B cells in GCs via autocrine and paracrine signaling. Similarly, elevated levels of IL-21 in the GC of ABMR recipients suggest its possible function in the GC during the onset of active rejection [9]. Blocking of IL-21 activity ameliorated the graft-versus-host disease after hematopoietic stem cell transplantation [28].

Intriguingly, IL-21 can also mediate immunosuppressive effect. Studies have reported that IL-21 induces apoptosis of immunocytes by upregulating the expression of transcription factor Bcl-2 [29, 30]. Chesneau et al. recently reported the function of IL-21-dependent GZMB^+^ B cells in maintaining immune tolerance in kidney transplant recipients. Moreover, they observed elevated secretion of IL-21 by T cells after stimulation by B cells in tolerant recipients [23]. Their findings were mainly based on *in vitro* experiments and lacked the study of serum cytokine levels in patients. In the present study, we measured the cytokine levels in kidney transplant recipients. Elevated levels of IL-21 were observed in patients with stable immune status. Serum IL-21 levels negatively correlated with serum Cr levels, whereas with an AUC of 0.822 (*P* < 0.001), IL-21 measurement did not outperform the diagnostic capacity of Cr (AUC: 0.989, *P* < 0.001).

IL-21 is involved in antibody class switching of B cells in both human and mouse systems to activate the humoral response. The IgG production level was reported to be slightly lower in IL-21R-deficient mice [31]. However, the blocking of both IL-4 and IL-21R severely impaired the IgG response [31]. In addition, we evaluated the rate of antibody class switching in patients post kidney transplantation; it was largely intact when acute rejection occurred. Because the serum IL-4 levels were comparable among NR, TCMR, and ABMR cohorts, this result was expected.

Patients with stable graft function for more than 5 years were enrolled in the NR cohort. In contrast, the average time of rejection in the total rejection group was 575.14±114.03 days. NR and rejection groups were not matched with respect to the time from transplantation due to insufficient follow-up durations to define NR. However, no correlation was observed between the time of rejection and cytokine and antibody levels (data not presented).

*IL-21* is a well-conserved gene [32]. The distribution of its effector IL-21R and the downstream signaling pathways, through which it regulates the differentiation and functions of lymphoid cells responsible for humoral response, are similar in human and mice [33]. Hence, we established a KT mouse model to further directly address the role of IL-21 in kidney transplantation. Exogenous IL-21 was injected into KT mice to mimic the serum IL-21 levels in immune stable patients. However, unexpectedly, the injection of exogenous IL-21 induced enhanced inflammatory infiltration, C4d deposit, and allograft injury including glomerulitis, tubulitis, and interstitial inflammation in the transplanted kidney. These findings suggest that exogenous IL-21 induced a higher rate of rejection instead of maintaining immune tolerance, a finding considerably different from that reported by previous studies [9, 22].

IL-21 exerts its effect on a variety of immune cells via the IL-21R to promote cell differentiation and maturation [33]. It is involved in the generation and differentiation of Tfh cells through an autocrine loop and amplifies its secretion [15, 27]. Moreover, it augments the proliferation, maturation, and differentiation of B cells by upregulating transcription factors such as Bcl-6 and Blimp-1 in the GC [13, 34]. In the present study, the administration of IL-21 led to the corresponding increased IL-21R expression in the spleen. The upregulated expression of IL-21R allows IL-21 to perform its growth factor-like functions via the IL-21/IL-21R signaling pathway. Furthermore, flow cytometry was used to analyze the proportion of immune cells in the peripheral blood and spleen of KT mice after IL-21 injection. An increase in the number of B cells in both peripheral blood and spleen was observed, accompanied by an elevated number of CD4^+^ T cells in the peripheral blood.

Tfh cells are the primary source of serum IL-21, whereas the ability of Tfh subsets to produce IL-21 is variable. Recent studies have reported that Tfh cells can be further divided into GC Tfh cells and peripheral Tfh cells based on different locations [35]. The IL-21 secretion ability of GC Tfh cells is extremely low such that little or no induction of IL-21 in antigen-specific GC Tfh cells was detected in macaque studies [36–38], whereas the peripheral Tfh cells have been reported to secrete high levels of cytokines such as IL-21 [39]. We detected decreased serum IL-21 levels in mice after KT despite the administration of exogenous IL-21, which was similar to the serum level of IL-21 in patients with acute rejection after KT, with a notably higher level of IL-21 in the spleen observed after IL-21 injection. Besouw et al. recently reported a correlation between increased donor-specific IL-21-producing cells, especially circulating Tfh cells, and kidney graft rejection [40]. Accumulation of Tfh cells in follicular-like structures of the transplanted kidney was reported by Graav et al. [41]. This could be attributed to the fact that during acute rejection onsets, peripheral IL-21-producing Tfh cells enter into the spleen and allograft, activating T and B cell proliferation and maturation by secreting IL-21 in the GC or follicular-like structures, leading to acute rejection.

The present study had certain limitations. First, the number of patients enrolled in this study was relatively low because of the low incidence rate of acute rejection in our kidney disease center. As healthy volunteers do not have alloantigens or take immunosuppressants, the immune status of such population is considerably different from that of kidney transplant recipients. Although our study could not include healthy controls due to their unwillingness to provide samples, we believe the NR cohort used as the disease control should be sufficient for studying transplant immunology changes in allograft rejection. This is because the immune status of the NR cohort is considerably closer to that of rejection cohorts. Second, the present study did not provide biopsy information of patients with stable allograft function because patients refused to undergo biopsy due to a Chinese tradition. Third, although we studied the critical function of IL-21 in kidney transplantation, the interpretation of results still requires more clinical evidence.

In conclusion, we observed distinctly lower levels of serum IL-21 in acute rejection patients, with a diagnostic and therapeutic implication of IL-21 from a precision medicine perspective. The mouse KT model proved useful in conducting *in vivo* studies of IL-21 biology. The injection of exogenous IL-21 triggered allograft rejection in kidney transplantation mice and resulted in enrichment of IL-21 in the spleen. Flow cytometry study revealed the proliferation of CD4^+^ T and CD19^+^ B cells in the peripheral blood and spleen when rejection occurred. These findings suggest the involvement of IL-21 in transplant immunobiology, highlighting its therapeutic potential in acute rejection.

## MATERIALS AND METHODS

### Patients

The study was approved by the Research Ethics Committee of The First Affiliated Hospital, College of Medicine, Zhejiang University (Hangzhou, China; approval number: 2016-512). All patients provided written informed consent. From September 2017 to September 2018, a total of 66 peripheral blood samples were collected from 66 patients after kidney transplantation, of which 30 were NR, 28 were acute TCMR, and 8 were active ABMR. Of the 28 patients in the TCMR group, 12 patients belonged to grade IA, two patients belonged to grade IB, 13 patients belonged to grade IIA, and 1 patient belonged to grade IIB. Patients with stable allograft function (creatinemia < 150 mmol/L and proteinuria < 1g/24 h [23]), negative panel reactive antibodies, and no BK virus infection for more than 5 years post transplantation were enrolled into the NR cohort. Patients with histological confirmation of acute rejection were enrolled in the rejection cohort, which was further divided into TCMR and ABMR according to the Banff 2017 criterion [24]. Exclusion criteria included acute infection, use of glucocorticoids, or anti-thymocyte globulin (ATG) pulse in 3 months. The characteristics of different subgroups are summarized in Table 1.

The peripheral blood samples of patients in the NR cohorts were collected during their routine follow-up investigations. The peripheral blood samples of patients in the acute rejection cohort were collected before renal biopsy and anti-rejection treatment. Histopathology was conducted by two experienced pathologists in our hospital.

### Detection of serum cytokine levels and isotype class switching in serum

Serum levels of cytokines IL-2, IL-4, IL-6, IL-10, IL-12, IL-17, IL-21, IL-27, IFN-γ, and TNF-α, and IgM and IgG subsets were measured by the multiplex bead immunoassay (HGAMMAG-301K-05, Millipore for cytokine detection; LXSAHM-10, R&D system for antibody detection, Shanghai Universal Biotech Co., Ltd) according to the manufacturer’s instructions. Briefly, the beads with standards and samples were incubated for 1 h at room temperature. The wells were washed with wash buffer and incubated with primary antibodies for 30 min. Afterward, the coated beads were incubated with biotinylated antibodies for 30 min. The analysis was performed using the Luminex system (X-200).

### Animals

All protocols involving animals were approved by the Institutional Animal Care and Utilization Committee of The First Affiliated Hospital, College of Medicine, Zhejiang University. The C57BL/6 and Balb/c mice were purchased from the Laboratory Animal Center of Zhejiang University. All mice were housed in sterile microisolator cages. Eight-week-old male mice were used in all experiments.

### Kidney transplantation and treatment

The left kidney from Balb/c mice was transplanted into C57BL/6 mice with anastomosis to the inferior vena cava and the recipient abdominal aorta, ureter anastomosis to the bladder as previously described [42]. The right kidney remained intact. Recombinant murine IL-21 (BMS6021, eBioscience; 10 μg in 100 μL PBS) (*n* = 9) [43] or 100 μL PBS (*n* = 7) was administered by tail vein injection into the recipients at days 0, 2, 4, and 6 after transplantation (Figure 5A). PBS was administered by tail vein injection into mice without transplantation (blank control, *n* = 3) simultaneously. All animals were sacrificed 7 days post-transplantation.

### Histopathological analysis

Primary renal, spleen, and renal allograft tissue cross-sections were fixed in 10% formalin and embedded in paraffin. Five μm sections were cut with a Leica CM1900 cryomicrotome (Leica Biosystems Division of Leica Microsystems Inc.). The sections underwent H&E, PAS, and Masson's staining for histological evaluation. Immunochemistry staining was performed following the manufacturer’s protocol. Briefly, sections were deparaffinized by successively washing them with xylene, 100% and 95% ethanol, and distilled water. After antigen unmasking and blocking, the sections were incubated with anti-C4d antibody (HP8033; Hycult Biotech, USA) or anti-IL21R antibody (ab5980; Abcam, UK) overnight at 4°C. The rabbit-specific HRP/AEC IHC Detection Kit Micro-polymer (Abcam, UK) was used to visualize the stained sections, followed by staining with diaminobenzidine and counterstaining with hematoxylin. The acute humoral rejection was confirmed by the presence of DSA, linear C4d staining in peritubular capillaries, and histologic evidence of graft injury [44]. A well-experienced pathologist scored the histopathologic characteristics of rejection, including tubulitis, glomerulitis, interstitial inflammation, intimal arteritis, and peritubular capillaritis according to the Banff lesion scores [45]. ImageJ software was used to analyze the captured images.

### DSA detection

The serum DSA titers in kidney transplant mice were measured using the previously reported method [46]. In brief, after pretreatment with mice Fc blocker, the cell suspensions of kidney donor spleen were incubated with diluted recipient serum collected on day 7 post kidney transplantation. The cells were washed using PBS thrice and subsequently incubated with anti-mouse IgG (Abcam) for 15 min on ice. The unbound antibody was washed away with PBS and the cells were analyzed by flow cytometry. The mean channel fluorescence of each sample was calculated using the FlowJo software (Tree Star Inc.) and reported as a titer.

### Cytometric bead array

Cytokines from the peripheral blood, spleen, and transplanted kidney were isolated as previously described [47]. Cytokine levels in the peripheral blood and spleen of mice after kidney transplantation were measured by cytometric bead array (CBA). The spleen and kidney tissues were mechanically homogenized, and the supernatants were collected. The Mouse Soluble Protein Master Buffer Kit and the appropriate CBA flex sets Mouse Th1/Th2/Th17 Cytokine Kit (BD Biosciences) were used to perform CBA analysis.

As per the manufacturer’s protocol, the supernatants were mixed with capture beads after 2× dilution in the diluent. Flow cytometry samples were run on ACSCantoII, LSRFortessa (BD Biosciences), and the data were analyzed using the FlowJo software (Tree Star Inc.). The median fluorescence intensity on beads was used for statistical analysis. A standard curve was used to extrapolate the protein concentrations.

### Isolation of cells from spleen, kidneys, and graft

The tissue was gently dissociated using the gentleMACS Dissociator (Miltenyi Biotec) in the PBS/bovine serum albumin (BSA)- ethylene diamine tetraacetic acid (EDTA) buffer. The buffer was prepared by mixing the BSA stock solution with autoMACS Rinsing Solution containing EDTA at a ratio of 1:20 (Miltenyi Biotec). The MACS Pre-Separation Filters (Miltenyi Biotec) were used to ensure that the dissociated tissue samples were free of cell clumps, promoting successful MACS separation and subsequent analysis.

### Flow cytometry

Peripheral blood was collected in sodium heparin tubes. Next, the peripheral blood mononuclear cells (PBMCs) were isolated using Ficoll (GE Healthcare) gradient centrifugation on the same day. CD4^+^ T cells and CD19^+^ B cells were analyzed by Flow cytometry. In brief, the PBMCs were labeled with anti-CD4 and anti-CD19 antibodies for 30 min at room temperature in the dark. Gates for CD4^+^ T cells and CD19^+^ B cells were determined based on the staining with anti-mice CD4 and anti-mice CD19 antibodies (BioLegend). The number of CD4^+^ T cells and CD19^+^ B cells in the peripheral blood and spleen of mice after KT was determined by FACSCalibur (BD Biosciences). FlowJo software (Tree Star Inc.) was used for further analysis.

### Statistical analysis

All statistical analyses were performed using the GraphPad Prism 8 (La Jolla). Continuous data are presented as mean±standard error of the mean (SEM), whereas frequencies are presented as proportions. One-way analysis of variance (ANOVA) was performed for multiple group comparisons. The Mann-Whitney *U* test was performed for unpaired *t* test. For categorical covariates, Chi-square tests were used to generate *P*-values. Linear regression was performed to determine correlations. All presented *P*-values were calculated with the two-tailed test; a *P*-value < 0.05 was considered significant.

## Supplementary Material

Supplementary Figures

Supplementary Table 1
