# Supplementary material for: Elevated serum IL-21 levels are associated with stable immune status in kidney transplant recipients and a mouse model of kidney transplantation
Source: Aging (Albany NY). 2020 Sep 29;12(18):18396–414. doi: 10.18632/aging.103713 (PMC7585127; doi:10.18632/aging.103713)
Supplement: Supplementary Figures [file aging-12-103713-s002..pdf]

SUPPLEMENTARY FIGURES

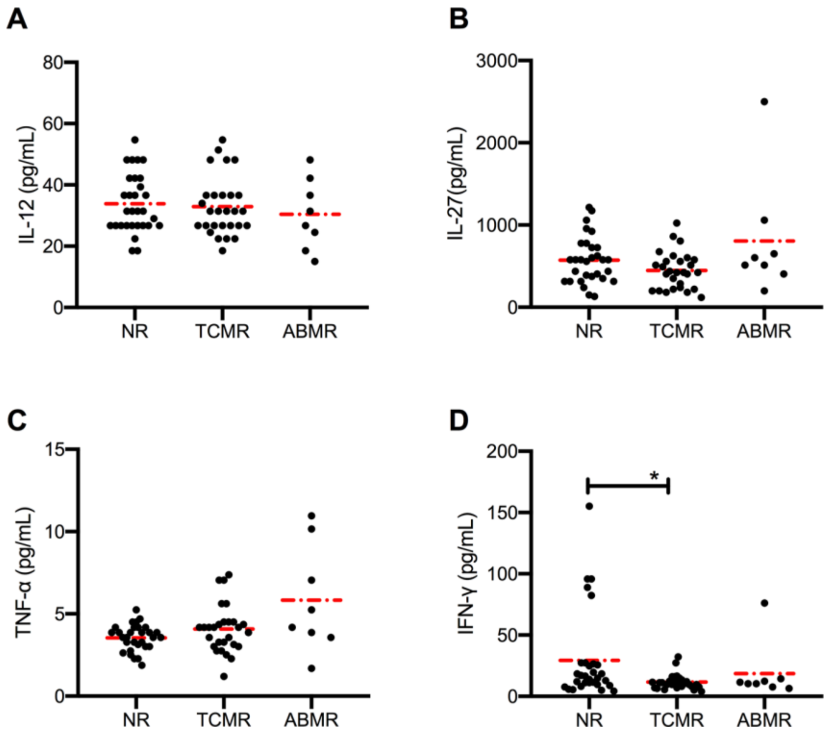

Supplementary Figure 1. Serum cytokine levels in transplant recipients. Serum levels of IL-12, IL-27, TNF-α, and IFN-γ (**A**–**D**) in patients with NR (n = 30), TCMR (n = 28), and ABMR (n = 8) cohorts were measured before the anti-rejection therapy. Red dash dot line represents the mean value. *$P < 0.05$ compared with NR cohorts. ABMR, antibody-mediated rejection; IFN, interferon; IL, interleukin; TNF, tumor necrosis factor; NR, non-rejection; TCMR, T cell-mediated rejection.

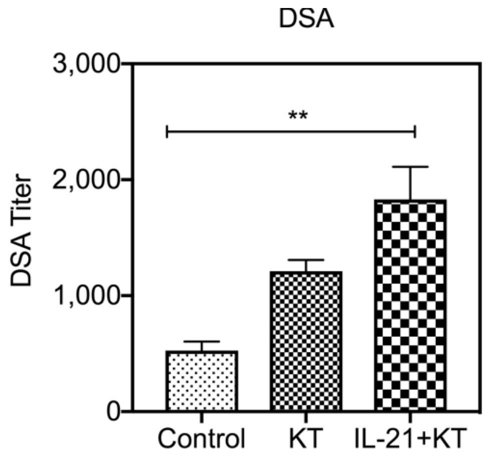

Supplementary Figure 2. Higher DSA titers were observed after IL-21 administration. **$P < 0.01$ compared with the control. DSA, donor-specific antibody; IL, interleukin; KT, kidney transplant.

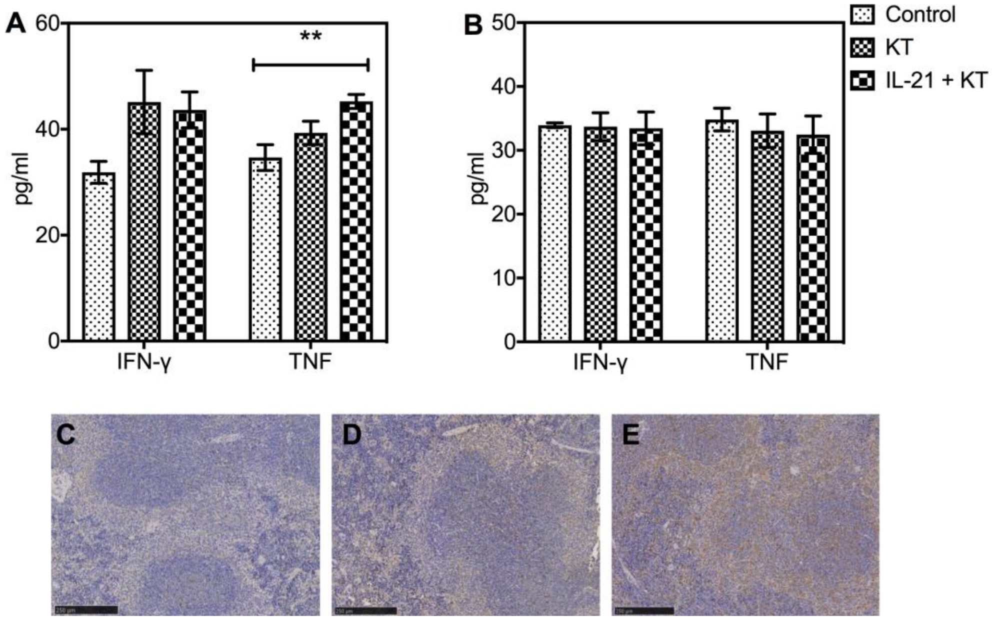

Supplementary Figure 3. Exogenous IL-21 alters cytokine and IL-21R expression in mice after kidney transplantation. The levels of IFN-γ and TNF-α in (**A**) the peripheral blood and (**B**) spleen on day 7 post-transplantation were measured by CBA. The expression of IL-21R in the spleen of (**C**) control, (**D**) KT, and (**E**) IL-21+KT mice on day 7 post-transplantation. Values represent the mean (±SEM). **P < 0.01 compared with the control. CBA, cytometric bead array; IFN, interferon; IL, interleukin; KT, kidney transplant; SEM, standard error of the mean; TNF, tumor necrosis factor.
