# Supplementary material for: Elevated serum IL-21 levels are associated with stable immune status in kidney transplant recipients and a mouse model of kidney transplantation
Source: Aging (Albany NY). 2020 Sep 29;12(18):18396–414. doi: 10.18632/aging.103713 (PMC7585127; doi:10.18632/aging.103713)
Supplement: Supplementary Table 1 [file aging-12-103713-s001..pdf]

SUPPLEMENTARY TABLE

Supplementary Table 1. Correlation between serum cytokine levels and creatinine levels at rejection onset.

	**IL-2**	**IL-4**	**IL-6**	**IL-10**	**IL-12**	**IL-17**	**IL-21**	**IL-27**	**IFN-γ**	**TNF-α**
Slope	0.04035	0.004171	0.002605	0.002559	0.001074	−0.0005405	−0.01824	0.1259	−0.03793	0.006161
P-value	0.5044	0.8906	0.1431	0.1787	0.9055	0.5304	0.0157	0.7046	0.1285	<0.0001
